# Nonsterol Triterpenoids as Major Constituents of *Olea europaea*

**DOI:** 10.1155/2012/476595

**Published:** 2012-03-20

**Authors:** Naïm Stiti, Marie-Andrée Hartmann

**Affiliations:** ^1^Institut de Biologie Moléculaire des Plantes du Centre National de la Recherche Scientifique (UPR 2357), Université de Strasbourg, 28 rue Goethe, 67083 Strasbourg, France; ^2^Institut für Molekulare Physiologie und Biotechnologie der Pflanzen (IMBIO), Universität Bonn, Kirschallee 1, 53115 Bonn, Germany

## Abstract

Plant triterpenoids represent a large and structurally diverse class of natural products. A growing interest has been focused on triterpenoids over the past decade due to their beneficial effects on human health. We show here that these bioactive compounds are major constituents of several aerial parts (floral bud, leaf bud, stem, and leaf) of olive tree, a crop exploited so far almost exclusively for its fruit and oil. *O. europaea* callus cultures were analyzed as well. Twenty sterols and twenty-nine nonsteroidal tetra- and pentacyclic triterpenoids belonging to seven types of carbon skeletons (oleanane, ursane, lupane, taraxerane, taraxastane, euphane, and lanostane) were identified and quantified by GC and GC-MS as free and esterified compounds. The oleanane-type compounds, oleanolic acid and maslinic acid, were largely predominant in all the organs tested, whereas they are practically absent in olive oil. In floral buds, they represented as much as 2.7% of dry matter. In callus cultures, lanostane-type compounds were the most abundant triterpenoids. In all the tissues analyzed, free and esterified triterpene alcohols exhibited different distribution patterns of their carbon skeletons. Taken together, these data provide new insights into largely unknown triterpene secondary metabolism of *Olea europaea*.

## 1. Introduction

Plant triterpenoids, which include sterols, steroids, and brassinosteroids, constitute a large and structurally diverse group of natural products, with over 100 different carbon skeletons [1, 2]. Oxidative modifications and glycosylations generate more chemical diversity [3]. Sterols and nonsterol triterpenoids are synthesized via the cytoplasmic acetate/mevalonate pathway and share common biosynthetic precursors up to (3*S*)-2,3-oxidosqualene (OS). The conversion of OS to cycloartenol by the cycloartenol cyclase (CAS, EC 5.4.99.8) is the first committed step in sterol biosynthesis, but OS can be also cyclized by distinct OS cyclases (OSCs), also known as triterpene synthases, into a variety of triterpene skeletons including those of *α*- and *β*-amyrins, the most commonly occurring plant triterpenes. These nonsterol triterpenoids are then metabolized into multioxygenated compounds, the precursors of triterpene saponins [4]. Thus, OS cyclization by the various triterpene synthases is a major branch point in the regulation of the carbon flux toward either the sterol pathway (primary metabolism) or the nonsterol triterpenoid pathway (secondary metabolism).

During these last ten years, triterpenoids isolated from a large number of plant organs from different species have been reported to exhibit a variety of antimicrobial, antioxidant, anti-inflammatory, antiviral, or antitumor-promoting biological activities [5–7]. Whereas the biological roles of sterols and brassinosteroids are well known [8, 9], the functions of nonsterol triterpenoids *in planta* still remain poorly understood. Nevertheless, there is increasing evidence that these “secondary” metabolites do contribute to plant defense, as attested by the production of triterpenic phytoalexins [10] or saponins [11] in response to biotic and abiotic stresses. Triterpenoids are known to be constituents of surface waxes on leaves and fruit of various species [12–15] and consequently affect cuticular structure and water permeability. These compounds play important roles in plant-insect interactions. As an example, Guhling et al. have mentioned the contribution of lupeol to the formation of epicuticular wax crystals at the surface of *Ricinus communis* stems, making them slippery [16]. These crystals serve as a physical barrier, hampering insect adhesion and preventing herbivores from climbing vertical plant organs. Triterpenoids have been also reported to be major components of the insect-trapping glue of *Roridula* species [17].

Olive (*Olea europaea* L.) tree cultivation started 6000 years ago on the Mediterranean shores from where it has continued to spread throughout many countries. Olive oil has become increasingly popular due to its advantageous nutritional and medicinal properties. However, while the beneficial effects of olive oil monounsaturated fatty acids and phenols are well recognized, little attention has been paid to minor compounds, including triterpenoids. In a previous work, we reported the composition of sterols and nonsteroidal triterpenoids as free and esterified conjugates in olive fruit, along with the changes that occurred throughout ripening [18]. In particular, we showed that mature olive fruit contained substantial amounts of oleanolic acid and maslinic acid, two triterpene acids known to protect humans against several diseases [19]. However, as these compounds are mainly located in the surface waxes of the olive skin [20], they occur only at low amounts in the oil. The present study provides a detailed investigation of the triterpenoid profile of other aerial organs (floral bud, leaf bud, stem, young and mature leaves) that were harvested from the same *O. europaea *tree as olive fruit used previously [18]. Evidence is given for the occurrence of a vast array of nonsterol triterpenoids in all the tissues analyzed, with a predominance of oleanane-type triterpenoids. The content of free and esterified sterols in the different tissues was also determined. Finally, attention was also paid to sterols and triterpenoids in callus cultures, a potential readily available source of bioactive compounds.

## 2. Material and Methods

### 2.1. Plant Material and Callus Tissue Cultures

The various organs (olive fruit, floral buds, leaf buds, stems, young and mature leaves) were all collected from the same olive tree (*Olea europaea* L., cv chemlali), during the spring of 2005. This tree was grown in the “El Intilaka” olive grove, located in the area of Beni Khalled (Le Cap Bon, Tunisia). The quantities (in fresh wt) of collected organs were the following: 92 floral buds (140.8 mg), 125 leaf buds (132.6 mg), 67 young leaves (5.3 g), 84 mature leaves (16.9 g), and stems (23.4 g). The term “stem” was used to designate a small branch (diameter less than 1 cm) from which leaves were removed. We designated young leaves those with a length between 1.5 and 2 cm and mature leaves those with a length higher than 3.0 cm.

Olive callus cultures were initiated as follows. Seeds from the same cultivar were provided by Prof. N. Drira (Faculté des Sciences, Sfax, Tunisia) and allowed to germinate in the dark on a 1/2 Murashige and Skoog (MS) medium containing agarose (8 g/L), gibberellin A_3 _(2 mg/L), and sucrose (20 g/L). After 10 to 15 days, germinated seeds were transferred to 1/2 MS medium with agarose (8 mg/L) and sucrose (40 g/L) and grown at 24°C under a photoperiod of 16 h light/8 h dark for 12 weeks. Callus cultures were generated from an axenic leaf collected from seedlings grown *in vitro*. The leaf was wounded slightly with a scalpel and put on a 1/2 agar MS medium supplemented with sucrose (20 g/L) and 2,4-dichlorophenoxyacetic acid (0.1 mg/L). After 2 to 3 weeks, induced callus tissues were transferred to 1/3 agar MS medium containing sucrose (20 g/L), 6-*Υ*-*Υ*-(dimethylallylamino)-purine (0.1 mg/L) and 1-naphtalene acetic acid (0.5 mg/L) and grown at 25°C in the dark. Callus tissues were subcultured every 2 weeks onto a fresh supplemented MS medium.

### 2.2. Extraction and Isolation of Triterpenoids

Fresh *Olea europaea *samples were grounded in liquid nitrogen before being lyophilized. Free and esterified sterols and nonsteroidal triterpenoids were isolated from the various tissues as reported previously [18]. Briefly, sterols and nonsteroidal triterpenoids were extracted from dry material by refluxing at 65°C with dichloromethane/methanol (2 : 1 by vol). Free and esterified sterols were separated by TLC (Merck 60F_254_, 0.25 mm) and analyzed according to [21]. Free pentacyclic and tetracyclic triterpenes were recovered with the fraction of 4,4-dimethylsterols and triterpene diols, as a polar band located below 4-demethylsterols. Sterols and triterpenoids released from ester conjugates by hydrolysis with 6% KOH in methanol were purified as above. All compounds were acetylated with acetic anhydride/pyridine. Mono- and diacetate derivatives of sterols and nonsterol triterpenoids were quantified by gas chromatography (GC) using a Varian model 8300 chromatograph equipped with a FID and a DB-1 or DB-5 capillary column (25 m × 0.32 mm i.d., 0.25 *μ*m thickness, J&W Scientific). The temperature program included a fast rise from 60 to 230°C (30°C/min), a slow rise from 230 to 280°C (2°C/min), and a plateau at 280°C for 10 min. Free cholesterol was used as an internal standard. Triterpene acids were isolated from the total lipid extract as described previously [18], converted into methyl esters by treatment with ethereal diazomethane at 0°C for 1 h, then acetylated to give acetoxy methylesters. Mono- and diacetoxy methylesters of nonsterol triterpenoids were analyzed by GC under similar conditions as above, but with oleanolic methylester as internal standard and a slightly modified temperature program (from 60 to 240°C at 40°C/min, then from 240 to 300°C at 2°C/min and a plateau at 300°C for 25 min). The amount of each compound was calculated as the ratio of the respective GC peak area to the internal standard (cholesterol or oleanolic methylester) peak area. Quantitative determinations correspond to means of three replicates ±SD.

Both sterol and nonsterol triterpenoids were identified by GC-mass spectrometry (MS) carried out on a 6890 Agilent gas chromatograph equipped with an on-column injector and a DB-5 (J&W Scientific) capillary column (30 m × 0.32 mm, i.d., 0.25 *μ*m thickness) coupled to an Agilent 5973 mass selective detector using electron impact at 70 eV. Compounds were identified by their relative retention time (RR_t_) in GC and MS fragmentation pattern in GC-MS.

**Scheme 1:**
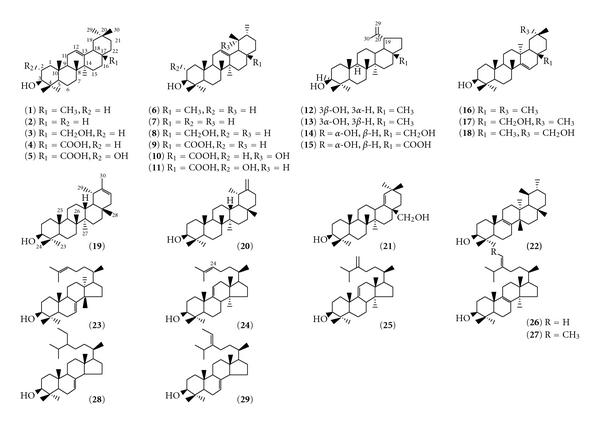
Structures of nonsterol triterpenoids cited in the paper.

RR_t_s were calculated in reference to that of the internal standard. Mass spectra were compared to those of available authentic samples or to literature data [18, 22, 23]. The structures of nonsterol triterpenoids described in the paper are shown in Scheme 1 and their MS fragmentation patterns are given in Table 1. 

## 3. Results

### 3.1. Free and Esterified Nonsterol Triterpenoids in *Olea europaea* Organs

#### 3.1.1. Identification of Free Nonsterol Triterpenoids

Analysis of free triterpenoids in the different aerial parts of the olive tree (Table 2) shows the occurrence of 18 pentacyclic triterpenoids that are distributed within 5 families of carbon skeletons: (i) oleanane type: *β*-amyrin **1 **(5*α*-olean-12-en-3*β*-ol), 28-nor-*β*-amyrin **2**, erythrodiol **3** (5*α*-olean-12-ene-3*β*,28-diol), oleanolic acid **4** (3*β*-hydroxyolean-12-en-28-oic acid), and maslinic acid **5** (2*α*,3*β*-dihydroxy-olean-12-en-28-oic acid); (ii) ursane type: *α*-amyrin **6** (5*α*-urs-12-en-3*β*-ol), 28-nor-*α*-amyrin **7**, uvaol **8** (5*α*-urs-12-ene-3*β*,28-diol), ursolic acid **9** (3*β*-hydroxyurs-12-en-28-oic acid), pomolic acid **10** (3*β*,19*α*-dihydroxyurs-12-en-28-oic acid) and 2-hydroxyursolic acid **11** (2*α*,3*β*-dihydroxyurs-12-en-28-oic acid); (iii) lupane type: lupeol **12** (5*α*-lup-20(29)-en-3*β*-ol), 3-*epi*-betulin **14** (5*α*-lup-20(29)-ene-3*α*,28-diol), and 3-*epi*-betulinic acid **15** (3*α*-hydroxy-5*α*-lup-20(29)-en-28-oic acid); (iv) taraxerane type: taraxerol **16** (taraxer-14-en-3*β*-ol) and myricadiol **17** (taraxer-14-ene-3*β*,28-diol or 28- taraxerol); (v) taraxastane type: Ψ-taraxasterol **19** (taraxast-20-en-3*β*-ol) and taraxasterol **20** (taraxast-20(30)-en-3*β*-ol). The structures of all these nonsterol triterpenoids are shown in Scheme 1. Among these triterpenoids, the compounds** 10**, **11**, **19**, and** 20 **had not been detected previously in the olive fruit [18]. Compounds **19** and **20** were identified as taraxastane triterpenes by their RR_t_ in GC and their MS fragmentation patterns [22, 23]. Compounds **10** and **11** appeared to be ursane-oxygenated derivatives. Acetylation of the methylester derivative of **10** yielded a monoacetate [24] that was recovered with the fraction of methoxy triterpene acid diacetates. The MS spectrum showed fragment peaks at *m/z* 528 instead of 570 (molecular peak) and 510 [M-H_2_O]^+^, suggesting the presence of a tertiary OH group. Retro-Diels-Alder (RDA) cleavage fragments from rings D and E gave prominent diagnostic peaks at *m/z* 260 [278-H_2_O]^+^, 219 [278-CO_2_Me]^+^, 201 [278-H_2_O–CO_2_Me]^+^, indicating the presence of a carboxyl methyl group on the C-28 and of an hydroxyl group located on the ring E. A peak at *m/z* 249, arising from RDA cleavage of rings A and B, was observed. This fragmentation pattern is consistent with MS data reported by Hidaka et al. [25] for ilexgenin A, a C-24 carboxylic derivative of pomolic acid, suggesting that compound **10** is 3*β*,19*α*-dihydroxy-12-urs-en-28-oic acid or pomolic acid. This triterpene acid was identified recently in *O. europaea* cell suspension cultures [26]. The MS fragmentation pattern of compound **11** was similar to that of methyl maslinic acid diacetate **5**, with abundant ions at *m/z* 262 and 203. These ion fragments usually result from the typical RDA cleavage of ring C of ursan-12-enes and olean-12-enes with a C-17 methoxycarbonyl and no hydroxyl groups on rings D/E [22]. However, in contrast to **5**, compound **11 **exhibited the most prominent ion at *m/z* 262 (base peak) instead of *m/z* 203. Furthermore, the appearance of a signal at *m/z* 249 and 189, resulting from successive losses of acetate from the ion at *m/z* 309, indicated that the additional oxygen atom was present on rings A/B. Compound **11** was identified as 2-hydroxyursolic acid. The stereochemistry at C-2 remained undetermined, but by analogy with maslinic acid (**5**), the corresponding oleanane triterpene acid possessing a 2*α*-OH, the structure of compound **11** as 2*α*,3*β*-dihydroxy ursolic acid or corosolic acid can be reasonably proposed. Moreover, this compound was also recently found in *O. europaea *cell suspensions [26].

The representative compound of each group of pentacyclic triterpenoids appeared to be converted into more highly oxygenated compounds such as diols, mono- and dihydroxylated triterpene acids. Through several oxidations steps, the C-28 methyl group is converted sequentially into a hydroxymethyl and a carboxylic group, but introduction of hydroxyl groups at other positions of the pentacyclic carbon skeleton also seems to take place. The enzymes involved in these oxidation reactions have not been characterized, but are likely cytochrome P-450 monooxygenases [27, 28]. The first identification of a P450 involved in the 24-hydroxylation of two pentacyclic triterpenes, *β*-amyrin and sophoradiol, has been reported recently [29], but the absence of hydroxylation at the C-17 of these compounds suggests that distinct P450 oxidases might be needed to hydroxylate different carbon positions.

No triterpene acids with a taraxerane or taraxastane skeleton were found.

More polar compounds with additional hydroxyl (i.e., triols) or carboxyl groups such as rotundic acid, tormentic acid, or 19*α*-hydroxyasiatic acid have been recently identified from *O. europaea* cell suspensions [26]. These compounds that were not looked for might occur in the olive tree organs as well.

#### 3.1.2. Quantification of Free Nonsterol Triterpenoids

Table 2 shows the distribution of free triterpenoids, as well as their content within the various parts of the olive tree. Oleanane-, ursane-, lupane-, and taraxerane-type pentacyclic triterpenoids were represented in all the organs, but taraxastane-type compounds, Ψ-taraxasterol **19** and taraxasterol **20**, occurred only in leaves. Oleanane-derived triterpenoids corresponding to *β*-amyrin **1 **and its oxygenated metabolites were by far the major nonsterol triterpenoids found. This pattern holds true especially for floral buds and mature olive fruit, where oleanolic acid **4 **and maslinic acid **5 **constituted up to 98-99% of total triterpenoids. Ursane-type triterpenoids were the second most abundant triterpenoids in the majority of organs. For instance, in young leaves they amounted to 29% of total triterpenoids, with a substantial contribution of uvaol **8**. However in stems and floral buds, lupane-type triterpenoids were slightly more abundant than ursane-type triterpenoids. Taraxerane-type and taraxastane-type triterpenoids were minor compounds (less than 1%).

Floral and leaf buds were clearly the richest parts of the olive tree in terms of free triterpenoids with as much as 26.8 mg and 10.2 mg per g dry matter (2.7 and 1%, resp.), amounts consistent with those found in other triterpene-rich plant parts such as the plane bark or rosemary leaves [30]. In comparison, triterpenoids were far less abundant in stems (0.04% of dry wt). Olive fruit and leaves contained intermediate amounts (0.2 to 0.4%).

The wealth of triterpenoids in olive floral buds is consistent with literature data on the wide array of pentacyclic triterpenoids detected in flowers from various plant families [5, 31–33]. Floral buds also contained a substantial amount of 3-*epi*-betulinic acid **15**, far more than that in other organs.

A different distribution pattern of free triterpenoids was observed in leaf buds (Table 2). In particular, high levels in *α*- and *β*-amyrins were found, but only *β*-amyrin appeared to be significantly converted into triterpene acids, oleanolic acid **4** and maslinic acid **5**. In young and mature leaves as well as in young olive fruit, substantial amounts of 28-nor-*α*-amyrin **7** and 28-nor-*β*-amyrin **2** were detected. These compounds lack a methyl group at C-17. As previously discussed [18], these two triterpenes might arise from the decarbonylation *in planta* of the aldehydes, 3*β*-hydroxy-5*α*-urs-12-en-28 al and 3*β*-hydroxy-5*α*-olean-12-en-28 al, which are the likely biosynthetic intermediates involved during the conversion of the C-28 hydroxymethyl group into a C-28 carboxylic group. Such a decarbonylation reaction on these unstable aldehydes could be promoted by exposure of leaves to intensive summer sunlight [34]. Occurrence of high levels of oleanolic and maslinic acids in olive leaves is well known [35, 36], but that of the ursane-type triterpenoids, pomolic acid **10 **and 2-hydroxyursolic acid **11**, has not been reported yet. Taken together, these data confirm the occurrence of pentacyclic triterpene acids in olive leaves as well as in leaves from various other plants [30, 37, 38].

Stems contained a low amount of nonsterol triterpenoids (Table 2). Although oleananes were the major nonsterol triterpenoids, this part of the olive tree was found to have the highest relative level in lupane derivatives, 12.2% of total free triterpenoids versus 0.5 to 1.9% in other organs. The main lupane compound was 3-*epi*-betulin **14**. Lupeol **12** but not its 3-epimer **13** was detected. These data are consistent with the occurrence of lupane-type triterpenoids in tree bark [39, 40] and the predominance of trinorlupeol in *Arabidopsis* stems [14].

#### 3.1.3. Changes in Relative Content of Triterpenoid Classes throughout Leaf Development

An interesting observation concerns changes throughout leaf development in the ratio of oleanane- to ursane-type triterpenoids following stepwise oxidations (Table 3 and Supplementary Figure S1 available online at doi: 10.1155/2012/476595). At the level of precursors, the ratio *β*-amyrin **1 **to *α*-amyrin **6** was comprised between 0.7 and 1.0 for all leaf tissues, indicating the presence of substantial amounts of *α*-amyrin besides *β*-amyrin. At the level of diols, the value of the ratio erythrodiol **3** to uvaol **8 **was 8.6-fold higher for leaf buds, but remained unchanged for both young and mature leaves. At the level of triterpene acids, a dramatic increase in the ratio (oleanolic acid **4** + maslinic acid **5**) to (ursolic acid **9** + pomolic acid **10** + 2-hydroxyursolic acid **11**) was observed, especially in mature leaves (a 150-fold increase compared to triterpene precursors and diols). If we consider the amounts of 28-nor-*β*-amyrin **2** and 28-nor-*α*-amyrin **7** as an estimate of the content in leaf tissues of the corresponding oleanane- and ursane-type aldehydes, the value found for the ratio **2**/**7 **is intermediate between the corresponding values for diols and acids. These data emphasize clearly the progressive metabolization of *β*-amyrin into maslinic acid and its accumulation during leaf development, but also address the question of the fate of uvaol arising from *α*-amyrin oxidation. Given the very low amount of ursolic acid in mature leaves (Table 2), the diol uvaol might serve as a substrate for a specific glycosyltransferase involved in saponin biosynthesis. In oleanane-type saponins, the largest class of triterpene saponins, the C-17 position of the triterpene carbon skeleton is frequently occupied by a hydroxymethyl group [4]. A similar situation is likely to occur for ursane-type saponins. The presence of uvaol **8** after acid hydrolysis of a butanolic extract from young olive fruit, a process allowing recovery of triterpene aglycones from saponins, would support this hypothesis (N. Stiti, unpublished results). Surprisingly, at the best of our knowledge, no information about *O. europaea* triterpenoid saponins was available, although in Tunisia olive oil pomaces have been used for various domestic tasks such as linen washing or hand care for a very long time, indicating clearly their wealth in saponins (N. Stiti, personal remark).

#### 3.1.4. Identification and Quantification of Esterified Nonsterol Triterpenoids

The distribution of esterified triterpenoids within the different olive tree organs is shown in Table 4. Only triterpene alcohols were analyzed after their release from esters. Because of the nonavailability of standards, we did not look for 3-monoesters of triterpene diols. In all tissues, the same pentacyclic triterpenes, belonging to the five classes of carbon skeletons described previously, were found, but their amounts were much lower than those of the free forms. Moreover, their relative distribution among the various organs was completely different. Leaf buds were the richest organs in esterified pentacyclic triterpenes, with almost equal amounts of *α*-amyrin **6**, *β*-amyrin **1**, and lupeol **12**. A clear predominance of oleanane compounds was observed only in floral buds. In leaves, both 3*α* and 3*β*-epimers of lupeol were detected, with a predominance of the 3*α*-epimer **13**.

Leaves also possessed substantial amounts (60 to 66% of total esterified triterpenes) of tetracyclic triterpenes with euphane (butyrospermol **23**) and lanostane (parkeol **24** and 24-methylene-dihydroparkeol **25**) skeletons, parkeol being the major compound. These tetracyclic triterpenes had been found previously in olive fruit, but only between 21 and 30 weeks after flowering (WAF) [18]. Low amounts (1–3%) of taraxerol, taraxasterol, and Ψ-taraxasterol were detected in several organs (Table 4).

### 3.2. Free and Esterified Nonsterol Triterpenoids in Callus Cultures

Composition of free nonsterol triterpenoids in *Olea europaea* callus tissue cultures is shown in Table 5. Compared to organs of the olive tree, pentacyclic triterpenoids from these undifferentiated cells were of the same five classes of carbon skeletons (i.e., oleanane-, ursane-, lupane-, taraxerane-, and taraxastane-type), but were distributed differently. Moreover, higher amounts of tetracyclic triterpenes were present. Several new compounds were identified for both classes of triterpenoids.

The predominant pentacyclic triterpenoids remained the oleanane- and ursane-type triterpenoids, which were found at nearly equal amounts (Table 5), indicating that *O. europaea *undifferentiated cells produce more ursane-type triterpenoids than the differentiated cells. Callus cultures contained the same oleanane-type triterpenoids as those occurring in the olive tree samples, that is, *β*-amyrin **1**, erythrodiol **3**, oleanolic acid **4,** and maslinic **5** acid, but also a new compound **21 **in low amount (1.6%). The MS of **21** showed a fragmentation pattern typical of a Δ^18^-oleanene, with a prominent ion at *m/z* 203 (base peak) and similar to that of the diacetate of 5*α*-olean-18-ene-3*β*,28-diol or moradiol **21 **[41]. Moradiol is a metabolite of germanicol, which has been found in olive oil [42].

Among ursane-type triterpenoids, we recovered *α*-amyrin **6**, 28-nor-*α*-amyrin **7**, uvaol **8**, ursolic acid **9**, 2-hydroxyursolic acid **11**, and pomolic acid **10** (4.5%). The triterpene acids **9**, **10 **and **11** were also detected in *Olea europaea* cell suspensions [26]. We found traces of a new triterpene alcohol, with a MS fragmentation pattern very similar to that of bauerenol [22], but its relatively early RR_t_ in GC with a DB5 column (1.326), close to that of *α*-amyrin (RR_t_: 1.335), was indicative of a compound with a Δ^8^-double bond rather than a Δ^7^-double bond. Thus, this compound was tentatively identified as isobauerenol **22**.

The lupane-type triterpenoids from callus tissue cultures were represented by 3-*epi*-lupeol **13**, 3-*epi*-betulin **14**, and 3-*epi*-betulinic acid **15**. Two other compounds, with late RR_t_s in GC with a DB5 column (1.478 and 1.493, resp.), were recovered in the fraction of diacetates. The MS fragmentation patterns of both compounds were very similar to that of 3-*epi*-betulin. One of those compounds might correspond to betulin and the other to 3*β*,30-dihydroxylup-20(29)-ene (hennadiol), but these structures need to be confirmed.

Besides taraxer-14-ene-3*β*,28-diol **17**, we found a second compound exhibiting an MS with fragmentations characteristic of the Δ^14^-taraxerene series of triterpenes [22] and very alike that of 28-hydroxytaraxerol (myricadiol) diacetate [23]. The presence of peaks at *m/z* 269, 202, and 189 indicated no additional group on cycles A, B, or C. Prominent ion fragments were observed at *m/z* 344 (base peak), 329, 284, and 269, corresponding to RDA fragmentation with collapse of ring D and successive losses of a methyl group, acetic acid and both groups. At the same time, the intensities of fragments at *m/z* 189 [262-CH_2_OAc]^+^ and 202 *m/z* [262-AcOH]^+^, which are characteristic for rings C and E, were decreased significantly. As reported by Budzikiewicz et al. [22], similar relative intensity changes were observed between erythrodiol diacetate and 30-hydroxy-*β*-amyrin diacetate MS fragmentation patterns, when the CH_2_OAc group is shifted from C-17 to C-30 (compare Figures 3 and 4 of [22], resp.). Thus, the position of the second hydroxyl group in this new taraxerene derivative might be located at C-29 or C-30. According to NMR and X-ray diffraction data from [43], the equatorial C-29 position for the CH_2_OAc would be favored and consequently the structure of **18** was established as taraxer-14-ene-3*β*,29-diol, a rare taraxerol derivative identified in the root bark of *Hippocratea excelsa* [43].

In contrast to olive tree organs in which free nonsteroidal tetracyclic triterpenes were not detected (Table 2), callus cultures contained substantial amounts of lanostane-type triterpenoids (34% of total free triterpenoids). By GC-MS, we identified parkeol **24** and 24-methylene-24-dihydroparkeol (**25**) (Table 1), but also 24-methylene lanost-8-en-3*β*-ol **26** and (24*Z*)-24-ethylidene-lanost-8-en-3*β*-ol **27 **(Table 1, [44]). Parkeol was particularly abundant (Table 4). Two other lanostane triterpenoids with very late RR_t_s in GC (1.478 and 1.493, resp. with a DB5-column) were also found. The first compound gave an intensive molecular ion at *m/z* 484, while the second one, with a MW of 482, showed a prominent fragment at *m/z* 341, corresponding to the loss of an unsaturated side chain plus 2H (Table 1). For both compounds, the absence of fragments at *m/z* 287 [M^+^-side chain-D ring] and *m/z* 227 [M^+^-side chain-D ring-acetate] was consistent with the absence of a 14*α*-methyl group. The two compounds displayed MS fragmentation patterns very similar to those of 24-ethyl- and 24-ethylidenelophenol but with a 14 mass increment of all ions fragments, suggesting that these two lanostanes can be ascribed to 4,4-dimethylsterols with a Δ^7^-double bond, 4,4-dimethylstigmast-7-en-3*β*-ol (**28**), and 4,4-dimethylstigmasta-7, *Z*-24(24^1^)-dien-3*β*-ol (**29**), respectively.

Besides lanostane-type triterpenoids, callus cultures were also found to contain butyrospermol **23**, an euphane-type triterpene.

It should be noticed that in these callus cultures, butyrospermol and parkeol were present as free forms, while in all the olive tree organs, both compounds were present only as fatty acid conjugates.

Callus cultures also contained esterified triterpenoids (Table 5). As in the case of olive tree organs, only esterified triterpenoid precursors were analyzed. We identified at least 24 compounds distributed between six families of carbon skeletons: oleanane, ursane, lupane, taraxastane, euphane, and lanostane. The major constituents were parkeol **24 **and butyrospermol **23**, two tetracyclic triterpenes representing together 76% of total esterified triterpenes. The other triterpenoids found were *β*-amyrin **1**, 3-*epi*-lupeol **13**, and *α*-amyrin **6**.

### 3.3. Free and Esterified Sterols in the Various Organs of the Olive Tree

In all the olive tree organs analyzed, free sterols were present as end products as well as usual biosynthetic precursors, 4,4-dimethyl- (24-methylenecycloartanol and traces of cycloartenol) and 4*α*-methylsterols (obtusifoliol, cycloeucalenol, 24-methylene- and 24-ethylidene lophenol) (Supplementary Table S1). Sterol end products were largely predominant (97 to 99% of total free sterols).

All organs contained the same sterols, with sitosterol by far the major component (87 to 95% of total free sterols). This sterol profile was similar to that of the olive fruit [18] and olive oil [42, 45]. Flower buds, with rapidly dividing cells, were particularly rich in free sterols. However, the sterol content of floral buds was lower than that of mature fruit. Conversely, leaf buds appeared to have the lowest amount of free sterols among the organs sampled. Floral buds contained relatively higher contents of 24-methylcholesterol (6.6%) than the other olive organs (1 to 2.3%), suggesting active brassinosteroid synthesis in this organ [46].

Analysis of sterols recovered from ester conjugates showed that the various parts of the olive tree contained the same esterified sterols as the free forms (Supplementary Table S2). Sterol end products and their precursors, 4,4-dimethylsterols (24-methylene cycloartanol) and 4*α*-methylsterols (cycloeucalenol and 24-ethylidene lophenol), were found. Sitosterol was the major sterol (between 84 and 93% of total sterol esters). In all the tissues except leaf buds, esterified sterols were found in lower amounts than the free forms (Supplementary Tables S1 and S2).

### 3.4. Free and Esterified Sterols from Callus Cultures

Supplementary Table S3 indicates that undifferentiated olive cells synthesize the same sterol molecules as the olive tree organs. This similarity held true for both free and esterified sterols. Sitosterol remained the main sterol in callus. In the case of free sterols, substantial amounts of 4,4-dimethyl- and 4*α*-methylsterols were found, with sterol end products representing only 66% of total sterols. Such an accumulation of biosynthetic precursors may indicate a slow sterol metabolism in the callus tissue cultures compared to differentiated cells, although the total amount of sterols in these cultures is largely predominant compared to the sterol content of any olive tree organ (Supplementary Tables S1, S2 and S3). Moreover, in contrast to the situation in olive tree organs, nonsteroidal triterpenoids were not the major products of triterpene metabolism in callus cultures (Table 4 and supplementary Table S3).

## 4. Discussion

Both sterol and nonsteroidal pathways share common biosynthetic precursors up to oxidosqualene OS. Thus, the OS cyclization step appears as the site for a complex regulatory process between primary and secondary triterpene metabolisms. In *Olea europaea *fruit, we observed previously a preferential channeling of OS molecules towards one or the other pathway, depending on the stage of olive fruit development [18]. As expected, the present data indicate that all the parts of the olive tree contain significant amounts of free sterols, metabolites needed to sustain membrane biogenesis and plant growth [8, 9]. Besides sterols, *O. europaea* actively produces a vast array of nonsterol triterpenoids belonging to seven families of carbon skeletons, but oleanane-type triterpenoids predominate as in the majority of higher plants. Together with ursane-type compounds, oleanane-type triterpenoids comprised more than 97% of nonsterol triterpenoids in aerial parts of the *O. europaea* tree. In all the tissues analyzed, nonsterol triterpenoids were found at higher amounts than free sterols, suggesting that a large part of OS was dedicated to the nonsteroidal pathway, at least at the development stages of the various olive tree tissues we harvested. An opposite situation occurs in olive callus cultures, in which sterols were found to be 3-fold more abundant than nonsterol triterpenoids, represented mainly by lanostane-type and not oleanane-type triterpenoids (Table 4 and Supplementary Table S3). In that context, it would be interesting to investigate whether carbon flux toward these nonsterol triterpenoids could be upregulated following addition of an elicitor.

Arising from OS cyclization by the various OSCs, pentacyclic triterpenes are oxidized stepwise into triterpene diols and acids that accumulate. In olive fruit throughout ripening [18], we have shown that the main pathway leading to nonsterol triterpenoids starts with oxidation of the C-28 methyl group of *α*- and *β*-amyrine to give successively the corresponding dialcohols, uvaol and erythrodiol, and acids, ursolic acid and oleanolic acid, whereas introduction of additional hydroxyl groups at C-2 takes place later (Supplementary Figure S1). We assume that similar steps occur in other olive tree organs. *O. europaea*, oleanane-type triterpenoids are metabolized preferentially into triterpene acids compared to ursane-type compounds. Triterpene acids with a carboxyl group at C-28 might serve further as substrates for specific glycosyltransferases [28, 47, 48] to form triterpenoid saponins. As already pointed out, the biosynthetic pathway leading to triterpene saponins in *O. europaea* remains to be deciphered, but our data constitute a basis for further studies.

The occurrence of a vast array of nonsteroidal triterpenoids in *O. europaea* raises questions on the origin of such structural diversity. Many OSCs involved in OS cyclization are able to form simultaneously a large number of products, resulting from the stabilization of specific carbocationic intermediates. Other OSCs catalyze the formation of only one product. Thus, OSCs are often categorized as either “multifunctional” or accurate, but that concept has been questioned recently [49]. Arabidopsis can form as many as 35 triterpenes [49]. Although only two cyclases, CAS1 and LUP2, are sufficient to produce all the C30 triterpene alcohols that have been detected in Arabidopsis [50, 51], its genome encodes 13 homologs of triterpene synthases [50, 52]. Baruol synthase (BARS1) itself generates as many as 23 compounds, with baruol the predominant product [49].

Up to now, three *Olea europaea* OSCs have been isolated and characterized: OEW, a lupeol synthase [53], OEX, a cycloartenol synthase, and OEA, an amyrin synthase [26]. OEX catalyzes the first committed step involved in the sterol pathway, the cyclization of OS into cycloartenol, and is likely responsible for the constitutive synthesis of sterols expressed in all the parts of the olive tree. OEW is an accurate enzyme, able to form only lupeol, the precursor of lupane triterpenoids that were detected in all the olive tree organs, but were relatively more abundant in the stems (Table 2). OEA has been isolated from *O. europaea* cell suspension cultures initiated from leaf stalks [26]. When expressed in a yeast cell-free system, OEA was shown to be able to form not only *α*-amyrin **6**, but also *β*-amyrin **1**, butyrospermol **23**, and Ψ-taraxasterol **19**. The major product was by far *α*-amyrin (60%). This multifunctional OSC might be responsible for the synthesis of these four pentacyclic triterpenes in olive tree leaves. The three isolated OSCs might not be sufficient to form all the triterpenoids detected in *O. europaea*. The large predominance of oleanane-type triterpenoids suggests that a *β*-amyrin synthase might be involved. We isolated two full-length cDNAs encoding two proteins comprising 760 amino acids and sharing 91% identity. These two proteins exhibited 86% identity with other plant *β*-amyrin synthases. We are characterizing the function of both genes in a lanosterol synthase-deficient *Saccharomyces cerevisiae* strain. The occurrence of a substantial amount of lanostane-type triterpenoids in callus tissue cultures also suggests the potential involvement of a lanosterol synthase in *O. europaea*, as already found in other plants [51, 54].

In conclusion, evidence is given here for the occurrence in all the parts of the *Olea europaea* tree analyzed of significant amounts of oleanane-type triterpenoids, among which oleanolic and maslinic acids are largely predominant. Although further work is needed to investigate their roles *in planta*, these two compounds already appear to be promising for their valuable effects on glucose and lipid metabolism as well as for their antimicrobial, antiviral, and antioxidant activities [19, 55]. In this context, because of their high content in both these triterpenic acids, waste olive pomaces as well as leaves collected with mechanically harvested fruit constitute potential sources for isolating these bioactive compounds and thus might contribute to better valorize the *Olea europaea* tree.

## Supplementary Material

In the Supplementary Material is given the qualitative and quantitative composition in free and esterified sterols of the various organs of *Olea europaea* tree and callus cultures.
Table S1: Amounts of free sterols in various organs of *Olea europaea* tree.
Table S2: Amounts of esterified sterols in various organs of *Olea europaea* tree.
Table S3: Amounts of free and esterified sterols in *Olea europaea* callus cultures.
Figure S1 represents a postulated biosynthetic pathway of nonsterol triterpenoids in *Olea europaea*.

## Figures and Tables

**Table 1:** GC-MS data of nonsterol triterpenoids.

Nonsterol triterpenoid	RR_t_ (DB-5)	MS fragmentation pattern
** (1) ** *β*-amyrin acetate	1.298	[50]
** (2)** 28-nor-*β*-amyrin acetate	1.288	[18]
** (3) **Erythrodiol diacetate	1.600	[22]
** (4) **Acetoxy oleanolic acid methylester	1.061	[18]
** (5) **Diacetoxy maslinic acid methylester	1.211	[18]
** (6) ** *α*-amyrin acetate	1.335	[50]
** (7) **28-nor-*α*-amyrin	1.317	[18]
** (8) **Uvaol diacetate	1.646	[42]
** (9) **Acetoxy ursolic acid methylester	1.092	[18]
** (10)** Acetoxy pomolic acid methylester	1.242	EIMS *m/z* (rel. int.): 528 [M]^+^, 510 [M-H_2_O]^+^ (5), 469 [M-60]^+^ (58), 453 [M-60-Me]^+^ (18), 409 (5), 396 (17), 260 (30), 249 (13), 219 (13), 201 (47), 190 (47), 189 (37), 179 (100), 146 (57), 133 (47)
** (11) **Diacetoxy 2-hydroxy- ursolic acid methylester	1.246	EIMS *m/z* (rel. int.): 570 [M]^+^, 511 [M-CO_2_Me]^+^ (5), 510 [M-60]^+^ (5), 450 [M-120]^+^ (7), 262 (100), 249 (17), 233 (7), 203 (77), 189 (22), 173 (11), 133 (49)
** (12) **Lupeyl acetate	1.340	[23]
** (13) **3-*epi*-lupeyl acetate	1.285	[18]
(**14) **3-*epi*-betulin diacetate	1.660	[18]
(**15) **Acetoxy 3-*epi*-betulinic acid methylester	1.042	[18]
(**16) **Taraxeryl acetate	1.280	[23]
(**17) **28-hydroxytaraxeryl diacetate	1.585	[22]
** (18) **29-hydroxytaraxeryl diacetate	1.667	EIMS *m/z* (rel. int.): 526 [M]^+^, 466 [M-60]^ +^ (22), 453 [M-73]^ +^ (13), 451 [M-60-Me]^ +^ (10), 406 [M-120]^ +^ (17), 355 (58), 344 (100), 329 (54), 316 (15), 284 (12), 269 (48), 255 (20), 229 (13), 216 (18), 202 (41), 189 (50), 177 (22)
(**19) **Ψ-taraxasteryl acetate	1.415	EIMS *m/z* (rel. int.): 468 [M]^+^ (19), 408 [M-60]^+^ (8), 393 [M-60-Me]^+^(8), 326 (18), 249 (7), 229 (8), 204 (12), 203 (13), 190 (42), 189 (100), 175 (27), 161 (17)
** (20)** Taraxasteryl acetate	1.423	EIMS *m/z* (rel. int.): 468 [M]^+^(17), 408 [M-60]^+^ (25), 365 (12), 299 (7), 249 (17), 218 (13), 204 (27), 203 (9), 189 (100), 175 (18), 161 (20)
** (21) **28-hydroxygermanicyl diacetate	1.617	EIMS *m/z* (rel. int.): 526 [M]^+^, 466 [M-60]^ +^ (28), 451 [M-60-Me]^ +^ (13), 406 [M-120]^ +^ (12), 393 (8), 276 (7), 249 (8), 221 (16), 216 [276-60]^ +^ (13), 203 [276-73]^ +^ (100), 189 (42), 187 (55), 175 (22), 133 (17), 119 (20)
** (22) **Isobauerenyl acetate	1.326	EIMS *m/z* (rel. int.): 468 [M]^+^ (33), 453 [M-Me]^+^ (14), 408 [M-60]^+^ (7), 393 [M-60-Me]^+^ (22), 301 (17), 289 (89), 255 (10), 241 (20), 229 (100), 215 (12), 205 (22)
** (23)** Butyrospermyl acetate	1.305	[18]
** (24) **Parkeyl acetate	1.348	[18]
** (25) **24-methylene lanost 9(11)-en-3*β*-yl acetate	1.381	[18]
** (26) **24-methylene lanosteryl acetate	1.341	EIMS *m/z* (rel. int.): 482 [M]^+^ (42), 467 [M-Me]^+^ (88), 407 [M-60-Me]^+^ (62), 383 [M-84-Me]^+^ (8), 323 [M-84-Me-60]^+^ (17), 301 [M-sc-56]^+^ (18), 283 (13), 255 (15), 241 (27), 215 (20), 95 (53), 69 (100)
** (27) **(24*Z*)-24-ethylidene lanosteryl acetate	1.44	EIMS *m/z* (rel. int.): 496 [M]^+^ (37), 481 [M-Me]^+^(100), 453 [M-43]^+^ (7), 421 [M-60-Me]^+^ (78), 398 [M-C_7_H_14_]^+^ (10), 383 [M-C_7_H_14_-Me]^+^ (23), 323 (27), 315 (13), 301 (13), 283 (40), 255 (38), 241 (20)
** (28) **4,4-dimethyl-5*α*-stigmast-7-en-3*β*-yl acetate	1.478	EIMS *m/z* (rel. int.): 484 [M]^+^ (100), 469 [M-Me]^+^ (13), 424 [M-60]^+^ (10), 409 [M-60-Me]^+^ (17), 384 (8), 343 [M-sc]^+^ (7), 341 [M-sc-2H]^+^ (17), 301 (4), 283 (53), 268 (3), 257 (10), 241 (20)
** (29) **4,4-dimethyl-5*α*-stigmasta-7, * Z*-24(24^1^)-dien-3*β*-yl acetate	1.493	EIMS *m/z* (rel. int.): 482 [M]^+^ (3), 467 [M-Me]^+^ (3), 422 [M-60]^+^ (2), 407 [M-60-Me]^+^ (5), 384 [M-98]^+^ (48), 369 (6), 341[M-sc-2H]^+^ (100), 324 (5), 309 (8), 283 (7), 281 (11), 255 (7), 241 (10)

^a^RR_t_ (relative retention time) values are relative to cholesterol (retention time set to 1) for compounds (**1)** to (**3)**,(**6) **to(**8)**, (**12)** to (**14)**, and (**16) **to (**29) **and relative to oleanolic methyl ester for compounds (**4)**, (**5)**, (**9)** to (**11)**, and (**15).**

**Table 2:** Amounts of free nonsterol triterpenoids in various organs of *Olea europaea* tree.

	Floral bud		Stem		Leaf bud		Young leaf		Mature leaf		Young fruit^a^		Mature fruit^b^	
	*μ*g/g dry wt	%	*μ*g/g dry wt	%	*μ*g/g dry wt	%	*μ*g/g dry wt	%	*μ*g/g dry wt	%	*μ*g/g dry wt	%	*μ*g/g dry wt	%
Oleanane type														
*β*-amyrin (**1**)	17.7		9.5		658		75.7		75.8		139		4	
28-nor-*β*-amyrin (**2**)	7.3		4.2		8.4		39.6		48.3		74		nd	
Erythrodiol (**3**)	56		161.4		232		452		383		401		13	
Oleanolic Acid (**4**)	19495		126.3		5495		485		65		1257		929	
Maslinic acid (**5**)	6643		43.2		2474		459		2755		830		1502	

Total	** 26212**	97.8^c^	** 344**	76.4	**8867**	87	** 1511**	68.4	**3327**	85.9	**2701**	84.2	**2448**	99.1

Ursane type														
*α*-amyrin (**6**)	3.8		8.5		974		86.4		70.4		192		nd	
28-nor-*α*-amyrin (**7**)	nd		0.6		nd		10.8		11.8		25		nd	
Uvaol (**8**)	0.8		34.5		37.9		479		411		245		0.4	
Ursolic Acid (**9**)	170		3		92		37		3.5		6		4	
Pomolic Acid (**10**)	nd		0.3		nd		3.1		nd		nd		nd	
2-hydroxyursolic acid (**11**)	56		1.1		72.8		25.8		14.7		nd		nd	

Total	**231**	0.9	** 48**	10.7	**1177**	11.6	**642**	29.1	**511**	13.2	** 468**	14.6	**4.4**	0.2

Lupane type														
Lupeol (**12**)	3.5		7		traces		traces		traces		nd		nd	
3-*epi*-betulin (**14**)	4.6		40.9		19		13.1		10.4		11		0.8	
3-*epi-*betulinic acid (**15**)	355		6.8		114		27.1		8.2		21		14	

Total	** 363 **	1.3	** 55**	12.2	**132**	1.3	**40 **	1.8	**19**	0.5	** 32**	1.0	**15**	0.6

Taraxerane type														
Taraxerol (**16**)	0.4		0.2		1.5		0.7		0.6		nd		2	
28-hydroxytaraxerol (**17**)	8.2		1.2		11		11.3		11.8		8		1	

Total	**8.6**	<0.1	** 1.4 **	0.3	**12.3**	0.1	**12**	0.5	**12.4**	0.3	**8**	0.2	3	0.1

Taraxastane type														
Ψ-taraxasterol (**19**)	nd		nd		nd		2.6		2.3		nd		nd	
Taraxasterol (**20**)	nd		nd		nd		1.2		1		nd		nd	

Total							**3.8**	0.2	**3.3**	0.1				

**Total ** **amount**	**26820 ± 3310**		**450 ± 45**		**10190 ± 1530 **		**2210 ± 110 **		**3875 ± 195**		**3210 ± 100**		**2470 ± 150**	

^****a****^Picked at 12 WAF; ^b^picked at 30 WAF [18]; ^c^percentages of the different classes of carbon skeleton; nd: not detectable.

**Table 3:** Ratio of oleanane- to ursane-type triterpenoids following oxidation steps of the C-28 CH_3_ throughout olive tree leaf development.

	Leaf bud	Young leaf	Mature leaf
C28–CH_3 _: ratio **1/6**	0.7^a^	0.9	1
C28–CH_2_OH : ratio **3**/**8**	6	0.9	0.9
C28–CHO : ratio **2**/**7**	—	4	4
C28–COOH : ratio (**4**+**5**)/(**9**+**10**+**11**)	48	14	155

^a^Ratios were calculated from data of Table 2.

**Table 4:** Amounts of esterified triterpene alcohols in various organs from *Olea europaea* tree.

	Floral bud		Stem		Leaf bud		Young leaf		Mature leaf		Young fruit^a^		Mature fruit^b^	
	*μ*g/g dry wt	%	*μ*g/g dry wt	%	*μ*g/g dry wt	%	*μ*g/g dry wt	%	*μ*g/g dry wt	%	*μ*g/g dry wt	%	*μ*g/g dry wt	%
Oleanane type														
*β*-amyrin (**1**)	42	78.9	2.1	28.4	88	34.6	26	13.6	9.2	15.8	nd		21	77.8
Ursane type														
*α*-amyrin (**6**)	2.9	5.5	2.0	27.0	79	31.1	5.8	3.0	2.7	4.6	2.0	33.3	traces	
Lupane type														
Lupeol (**12**)	6.2	11. 7	3.3	44. 6	83	32. 7	6.7	3.5	1.1	1.9	nd		nd	
3-*epi*-lupeol (**13**)	nd		nd		nd		21	11.0	8.4	14.4	2.0	33.3	4	14.8
Taraxerane type														
Taraxerol (**16**)	0.2	0.4	nd		0.8	0.3	2.4	1.3	0.7	1.2	2.0	33.3	2	7.4
Taraxastane type														
Ψ-taraxasterol (**19**)	0.6	1.1	nd		3.4	1.3	2.5	1.3	0.8	1.4	nd		nd
Taraxasterol (**20**)	0.2	0.4	nd		nd		0.6	0.3	0.2	0.3	nd		nd
Euphane type													
Butyrospermol (**23**)	nd		nd		nd		49	25.7	14.2	24.4	traces		traces
Lanostane type														
Parkeol (**24**)	0.8	1.5	traces		nd		76	40	20.5	35. 3	nd		nd	
24-methylene-24-dihydroparkeol (**25**)	0.3	0.5	nd		nd		0.6	0.3	0.4	0.6	nd		nd	

Total amount	53 ± 6		7. 4 ± 0.6		254 ± 15		191 ± 3		58 ± 4		6.0 ± 0.8		27 ± 4	

^a,b^ See legend of Table 2; nd: not detectable.

**Table 5:** Amounts of free and esterified nonsterol triterpenoids in *Olea europaea* callus cultures.

	Free		Esterified	
	*μ*g/g dry wt	%	*μ*g/g dry wt	%
Oleanane type				
*β*-amyrin (**1**)	8.5		25.6	
Erythrodiol (**3**)	7.2			
Moradiol (**21**)	0.8			
Oleanic acid (**4**)	45			
Maslinic acid (**5**)	14.5			

Total	** 76**	22	**25.6**	12.9

Ursane-type				
*α*-amyrin (**6**)	15.4		5.2	
28-nor-*α*-amyrin (**7**)	1.6			
Isobauerenol (**22**)	Traces			
Uvaol (**8**)	13			
Ursolic acid (9)	34			
Pomolic acid (10)	4.5			
2-hydroxyursolic acid (**11**)	9.5			

Total	** 78**	22.5	**5.2**	2.6

Lupane type				
3-*epi-*lupeol (**13**)	nd		16.0	
3-*epi-*betulin (**14**)	2.0			
Betulin + unknown betulin derivative	39			
3-*epi-*betulinic acid (**15**)	6.3			

Total	**47.3**	13.7	**16.0**	8.1

Taraxerane type				
Taraxer-14-ene-3*β*,28-diol (**17**)	3.2			
Taraxer-14-ene-3*β*,29-diol (**18**)	12.5			

Total	**15.7**	4.5	nd	

Taraxastane type				
Ψ-taraxasterol (**19)**	1.3		0.3	
Taraxasterol (**20**)	1.1			

Total	**2.4**	0.7	**0.3**	0.1

Euphane type				
Butyrospermol (**23**)	**9.2**	2.7	**51**	25.7

Lanostane type				
Cycloartanol	Traces		nd	
Parkeol (**24**)	39.5		99	
24-methylene-24-dihydroparkeol (**25**)	15.2		1.5	
24-methylene-24-dihydrolanosterol (**26**)	27		nd	
(24*Z*)-24-ethylidene-dihydrolanosterol (**27**)	3.1		nd	
4,4-dimethyl-5*α*-stigmast-7-en-3*β*-ol (**28**)	6.0			
4,4-dimethyl-5*α*-stigmasta-7,24*Z*(24^1^)-dien-3*β*-ol (**29**)	19.3			
Unknown lanostane derivatives	7.4		nd	

Total	**117.5**	33.9	** 101**	50.6

**Total amount **	**345 ± 40**		**200 ± 15**	

## Abbreviations

CAS: Cycloartenol synthase
OS: Oxidosqualene
OSC: Oxidosqualene cyclase
WAF: Weeks after flowering.
