# Supplementary material for: Nonsterol Triterpenoids as Major Constituents of Olea europaea
Source: J Lipids. 2012 Mar 20;2012:476595. doi: 10.1155/2012/476595 (PMC3317172; doi:10.1155/2012/476595)
Supplement: Supplementary file 1 — In the Supplementary Material is given the qualitative and quantitative composition in free and esterified sterols of the various organs of Olea europaea tree and callus cultures. Table S1: Amounts of free sterols in various organs of Olea europaea tree. Table S2: Amounts of esterified sterols in various organs of Olea europaea tree. Table S3: Amounts of free and esterified sterols in Olea europaea callus cultures. Figure S1 represents a postulated biosynthetic pathway of nonsterol triterpenoids in Olea europaea. [file 476595.f1.pdf]

Table S1
Amounts of free sterols in various organs of *Olea europaea* tree

	floral bud	stem	leaf bud	young leaf	mature leaf	young fruit[a]	mature fruit[b]
**4.4-dimethylsterols**							
cycloartenol	nd	nd	nd	nd	nd	nd	31
24-methylenecycloartanol	2.7	3	2.9	2.6	2.5	nd	255
**4α-methylsterols**	0.5	5.4	4.9	1.2	1.2	traces	24.9
**4-demethylsterols**							
brassicasterol	2.9	0.3	traces	traces	traces	1	0
24-methylcholesterol	43.3	4.6	2.7	2.1	1.6	3.8	19.4
stigmasterol	11.7	4.8	0.9	5.6	4.6	3.6	7.7
$\Delta^{5,23}$-stigmastadienol	0.2	0.1	0.1	0.1	traces	nd	nd
clerosterol	2.8	1.8	0.5	1.2	1	1.8	6
sitosterol	590	189	37.2	197	143	237	588
isofucosterol	0.8	0.2	0.5	0.3	traces	nd	26.2
**Total amount (μg/g dry wt)**	**655 ± 37**	**210 ± 15**	**50 ± 6**	**210 ± 15**	**155 ± 8**	**250 ± 22**	**960 ± 54**

[a] Picked at 12 WAF (weeks after flowering); [b] Picked at 30 WAF [18]; nd: not detectable.

Table S2
Amounts of esterified sterols in various organs of *Olea europaea* tree

	floral bud	stem	leaf bud	young leaf	mature leaf	young fruit[a]	mature fruit[b]
**4.4-dimethylsterols**							
cycloartenol	nd	nd	nd	nd	nd	nd	4.3
24-methylenecycloartanol	3	1.8	6.7	6.3	3	2	31.5
**4α-methylsterols**							
obtusifoliol	18.2	7.5	22.1	21.4	11.5	0.2	2.1
24-methylenelophenol	1.8	0.2	nd	nd	nd	1.2	1.1
cycloeucalenol	0.7	0.1	0.4	9.1	nd	0.7	1.9
24-ethylidenelophenol	1.5	nd	0.3	0.9	nd	0.5	9.9
**4-demethylsterols**							
24-methylcholesterol	9.6	0.9	3.4	2.2	0.7	1	7
stigmasterol	2.4	0.6	3.8	5.7	1.8	3.2	2.2
$\Delta^{5,23}$-stigmastadienol	0.1	traces	0.2	0.2	nd	nd	nd
clerosterol	0.8	0.3	1	1.2	0.3	0.3	2
sitosterol	83	36.6	94	117	39.4	27.3	188
isofucosterol	1.6	0.6	1.8	4.7	1.2	7.1	12.3
**Total amount (*µ*g/g dry wt)**	**125 ± 15**	**50 ± 6**	**135 ± 15**	**170 ± 15**	**60 ± 7**	**45 ± 5**	**260 ± 24**

[a,b] see legend of table S1.

Table S3
Amounts of free and esterified sterols in *Olea europaea* callus cultures

	**Free sterols**	**Esterified sterols**
**4.4-dimethylsterols**		
cycloartanol	14[a]	
24-methylene cycloartanol	84	9.4
Total	**98**	**9.4**
**4α-methylsterols**		
obtusifoliol	16	5.4
24-methylenelophenol	32	3.8
24-methyllophenol	11.4	6.8
cycloeucalenol	6.5	2.6
24-ethyl-*E*-23-dehydrolophenol	10.5	0.4
24-ethyl lophenol	39	0.9
24-ethylidene lophenol	147	0.9
Total	**261**	**21**
**4-demethylsterols**		
24-methylcholesterol	25.1	12.8
stigmasterol	15.3	5.0
$\Delta^{5,23}$-stigmastadienol	0.74	3.8
clerosterol	8.6	5.1
sitosterol	729	324
sitostanol	6.6	
isofucosterol	3.9	8.8
$\Delta^{5,24}$-stigmastadienol	2.9	2.4
Δ^{7}-stigmastenol	2.9	
Δ^{7}-avenasterol	0.6	
Total	**796**	**362**
**Total amount** (μg/g dry wt)	**1155 ± 120**	**392 ± 47**
