# Supplementary material for: Nonsterol Triterpenoids as Major Constituents of Olea europaea
Source: J Lipids. 2012 Mar 20;2012:476595. doi: 10.1155/2012/476595 (PMC3317172; doi:10.1155/2012/476595)
Supplement: Supplementary file 2 [file 476595.f2.pdf]

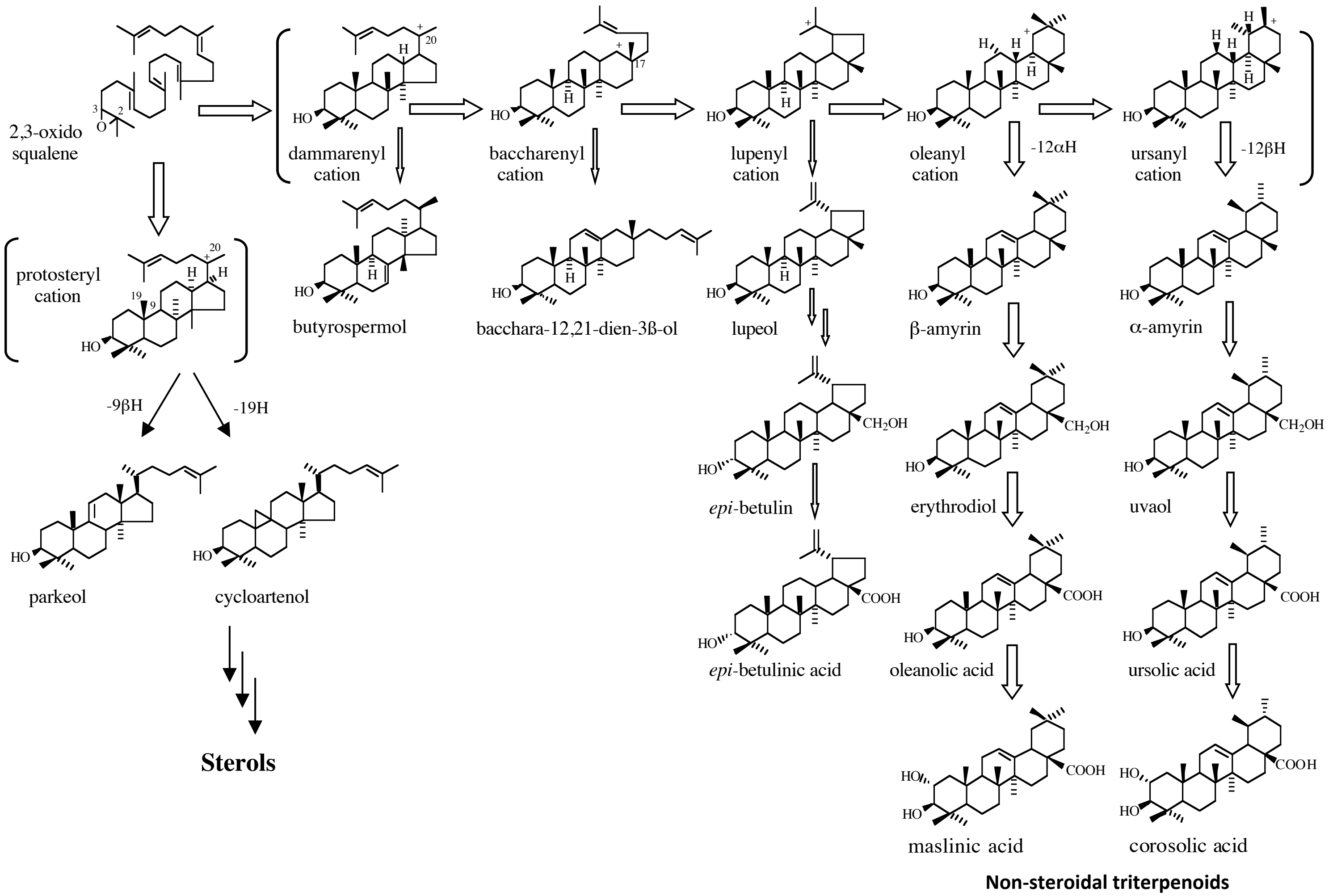

Figure S1 : Postulated biosynthetic pathway of nonsterol triterpenoids in *Olea europaea*

The width of the arrows is indicative of the relative proportions of the carbon flux between the different families of triterpenoids
